# phytochemdb: a platform for virtual screening and computer-aided drug designing

**DOI:** 10.1093/database/baac002

**Published:** 2022-01-28

**Authors:** Shafi Mahmud, Gobindo Kumar Paul, Suvro Biswas, Taheruzzaman Kazi, Shafquat Mahbub, Mohasana Akter Mita, Shamima Afrose, Ariful Islam, Sheikh Ahaduzzaman, Md. Robiul Hasan, Mst. Sharmin Sultana Shimu, Maria Meha Promi, Mobasshir Noor Shehab, Ekhtiar Rahman, Khaled Mahmud Sujon, Md. Wasim Alom, Anik Modak, Shahriar Zaman, Md. Salah Uddin, Talha Bin Emran, Md. Sayeedul Islam, Md. Abu Saleh

**Affiliations:** Microbiology Laboratory, Department of Genetic Engineering and Biotechnology, University of Rajshahi, Rajshahi 6205, Bangladesh; Microbiology Laboratory, Department of Genetic Engineering and Biotechnology, University of Rajshahi, Rajshahi 6205, Bangladesh; Microbiology Laboratory, Department of Genetic Engineering and Biotechnology, University of Rajshahi, Rajshahi 6205, Bangladesh; Department of Regenerative Dermatology, Graduate School of Medicine, Osaka University, Suita 565-0871, Japan; Department of Computer Science and Engineering, University of Rajshahi, Rajshahi 6205, Bangladesh; Department of Genetic Engineering and Biotechnology, University of Rajshahi, Rajshahi 6205, Bangladesh; Department of Genetic Engineering and Biotechnology, University of Rajshahi, Rajshahi 6205, Bangladesh; Department of Genetic Engineering and Biotechnology, University of Rajshahi, Rajshahi 6205, Bangladesh; Department of Computer Science and Engineering, University of Rajshahi, Rajshahi 6205, Bangladesh; Department of Genetic Engineering and Biotechnology, University of Rajshahi, Rajshahi 6205, Bangladesh; Department of Genetic Engineering and Biotechnology, University of Rajshahi, Rajshahi 6205, Bangladesh; Department of Genetic Engineering and Biotechnology, University of Rajshahi, Rajshahi 6205, Bangladesh; Department of Genetic Engineering and Biotechnology, University of Rajshahi, Rajshahi 6205, Bangladesh; Department of Genetic Engineering and Biotechnology, University of Rajshahi, Rajshahi 6205, Bangladesh; Department of Genetic Engineering and Biotechnology, University of Rajshahi, Rajshahi 6205, Bangladesh; Department of Genetic Engineering and Biotechnology, University of Rajshahi, Rajshahi 6205, Bangladesh; Department of Computer Science and Engineering, University of Rajshahi, Rajshahi 6205, Bangladesh; Microbiology Laboratory, Department of Genetic Engineering and Biotechnology, University of Rajshahi, Rajshahi 6205, Bangladesh; Microbiology Laboratory, Department of Genetic Engineering and Biotechnology, University of Rajshahi, Rajshahi 6205, Bangladesh; Department of Pharmacy, BGC Trust University Bangladesh, Chittagong 4381, Bangladesh; Department of Biological Sciences, Graduate School of Science, Osaka University, Machikaneyama-cho 1-1, Toyonaka, Osaka 560-0043, Japan; Microbiology Laboratory, Department of Genetic Engineering and Biotechnology, University of Rajshahi, Rajshahi 6205, Bangladesh

## Abstract

The phytochemicals of medicinal plants are regarded as a rich source of diverse chemical spaces that have been used as supplements and alternative medicines in the millennium. Even in this era of combinatorial chemical drugs, phytomedicines account for a large share of the statistics of newly approved drugs. In the field of computational aided and rational drug design, there is an urgent need to develop and build a useful phytochemical database management system with a user-friendly interface that allows proper data storage, retrieval and management. We showed ‘phytochemdb’, a manually managed database that compiles 525 plants and their corresponding 8093 phytochemicals, aiming to incorporate the activities of phytochemicals from medicinal plants. The database collects molecular formula, three-dimensional/two-dimensional structure, canonical SMILES, molecular weight, no. of heavy atoms, no. of aromatic heavy atoms, fraction Csp3, no. of rotatable bonds, no. of H-bond acceptors, no. of H-bond donors, molar refractivity, topological polar surface area, gastrointestinal absorption, Blood–Brain Barrier (BBB) permeant, P-gp substrate, CYP1A2 inhibitor, CYP2C19 inhibitor, CYP2C9 inhibitor, CYP2D6 inhibitor, CYP3A4 inhibitor, Log Kp, Ghose, Veber, Egan, Muegge, bioavailability scores, pan-assay interference compounds, Brenk, Leadlikeness, synthetic accessibility, iLOGP and Lipinski rule of five with the number of violations for each compound. It provides open contribution functions for the researchers who screen phytochemicals in the laboratory and have released their data. ‘phytochemdb’ is a comprehensive database that gathers most of the information about medicinal plants in one platform, which is considered to be very beneficial to the work of researchers on medicinal plants. ‘phytochemdb’ is available for free at https://phytochemdb.com/.

## Background

Nature has given the world of medicinal use an unprecedented but under-appreciated blessing, namely medicinal plants. Medicinal plants are full of potential pharmacological properties and can satisfy people’s desire to develop new medicines and treatments to deal with ancient diseases (1). Even now, in the era of synthetic drugs and combinatorial chemistry, phytomedicine still plays a pivotal role in the health management system (2). According to the World Health Organization, ∼80% of some Asian and African countries predominantly use traditional phytomedicine in their primary health issues (http://www.who.int/mediacentre/factsheets/fs134/en/). Twenty percent of the US population exercises herbal medicines as well (3). Phytomedicines worth 60 billion US dollars in the global market and its increment are indeed enviable (http://www.who.int/mediacentre/factsheets/fs134/en/).

Medicinal plants are considered to be the main natural reservoir for the invention and development of novel therapeutic molecules (4). These molecular structures evolved through evolutionary pressure (1). Therefore, it provides diversified properties (organosulfur compounds, limonoids, lignans, furyl compounds, alkaloids, polyenes, thiophenes, proteins, peptides, flavonoids, terpenoids, polyphenolics, coumarins and saponins) that are consistent with the lead structure in drug discovery (4). Phytochemicals with biological activity can bind to receptors of particular disease-specific molecular targets (1). This special feature can be used for virtual screening and other aspects of drug design (2). The huge pharmaceutical benefits and therapeutic effects of phytochemicals in medicinal functions have led to the production of many commercial and Food and Drug Administration (FDA)-approved drugs, which are used as antioxidant, antimicrobial, anti-inflammatory, anti-carcinogenic and anti-diabetic (5, 6). For instance, apomorphine (Parkinson) (7), arteether (malaria) (8), galantamine (Alzheimer) (9), nitisinone (hepatorenal tyrosinemia) (10), paclitaxel (cancer) (11), tiotropium (asthma and Chronic Obstructive Pulmonary Disease (COPD)) (12) and other phytomedicines recuperate various human maladies.

In addition, levomilnacipran and vortioxetine are used as antidepressants, while alpiropride is used as an anti-migraine drug. Furthermore, diabetes 2 caused by luseogliflozen, cystic fibrosis caused by ivacaftor, erythropoiesis caused by rh-erythropoietin-alfa, hemophilia A caused by susoctocog alfa, insomnia caused by suvorexant and osteoporosis caused by strontium ranelate are an example of comprehensive medication. In addition, azilsartan medoxomil is used as an antihypertensive drug, and amezinium methylsulfate is used as an antihypotensive drug. As revealed, antiviral phytochemicals including HEV-239 against hepatitis E, peramivir against H1N1 and cobicistat against HIV have shown reasonable efficacy in the treatment of a variety of viral diseases (13). Furthermore, edible macrophytes have long been a valuable source of traditional medicine, and freshwater macrophytes exhibit anticancer and antioxidant properties (14). Camptothecin, a terpene indole alkaloid isolated from the *Camptotheca acuminate*, demonstrated anti-tumor potential and was eventually licensed by the FDA for various types of cancer (15). Another phytochemical, luteolin (16), was approved for the inhibition of chronic inflammation, while quercetin (17) demonstrated strong anti-inflammatory properties. As a result, several medicinal compounds from the twentieth century were derived from phytochemicals such as aspirin, digoxin and quinone (18).

An appropriate database on medicinal plants and their related natural products, as well as their chemical structures and a repository of pharmacological and physicochemical information, is a major need, which can greatly aid drug development (19). In this direction, there are already several natural product databases focusing on phytochemistry such as CVDHD (20), KNAPSACK (21), Nutrichem (22), Phytochemica (1), TCMID (23), TCM@Taiwan (24), TCM-Mesh (25) and MAPS (26). However, these resources provide basic allocations. For example, some provide only downloadable convenience facilities, some offer virtual screening but do not accurately screen medicinal plant extracts, a few databases provide dockable libraries and some lack relevant phytochemistry (4). Therefore, a wide range of medicinal phytochemicals remain elusive for researchers (19). Furthermore, database resources are occasionally inadequate; for example, many contemporary databases contain downloaded phytochemicals, but researchers must utilize another site or tools to predict pharmacological properties. For example, the KNAPSACK and TCMID databases contain a large number of phytochemicals but lack chemical and pharmacological properties. As a result, any researcher can use this database to track the evolution and inclusion of new phytochemicals data in the future. And the database’s review team will assess the additional data’s quality before approving it alongside the existing phytochemical data. This will allow the database to expand at a faster rate, allowing it to become a one-of-a-kind repository of phytochemical data and information.

Therefore, we proposed a manually managed database, phytochemdb.com, which provides a comprehensive collection of plant-derived phytochemicals and their chemical characteristics, which can facilitate future research in computer-aided drug design, virtual screening, lead optimization and molecular docking. Currently, the database compiles about 8093 phytochemicals from 525 plants and the number will increase rapidly. This database integrates the molecular formula, three-dimensional (3D)/two-dimensional (2D) structure, canonical SMILES, molecular weight, no. of heavy atoms, no. of aromatic heavy atoms, fraction Csp3, no. of rotatable bonds, no. of H-bond acceptors, no. of H-bond donors, molar refractivity, topological polar surface area (TPSA), gastrointestinal (GI) absorption, BBB permeant, P-gp substrate, CYP1A2 inhibitor, CYP2C19 inhibitor, CYP2C9 inhibitor, CYP2D6 inhibitor, CYP3A4 inhibitor, Log Kp, Ghose, Veber, Egan, Muegge, bioavailability scores, pan-assay interference compounds (PAINS), Brenk, Leadlikeness, synthetic accessibility, iLOGP and Lipinski rule of five with the number of violations for each compound.

Anyone can retrieve the data of phytochemical substances through various search options, such as unique accession number (Phytochem ID), canonical SMILES, molecular formula and plant names. By simply searching for the plant name, one can find the phytochemicals obtained from each plant. This plant-derived phytochemical database is publicly available and is intended for use by the scientific community. ‘phytochemdb’ plans to expand in the future, so researchers who have screened phytochemicals in their laboratories and have published their data will be considered in this database. Thereafter, the upload option will enable researchers to contribute by uploading the 3D structure of the compounds in the database and provide the same accession number for the newly uploaded compound after passing the review process.

Finally, a structured dataset with precise phytochemical structure will become a powerful tool in the in silico drug design sector and will substantially aid in the identification of drug molecules from medicinal plants (27). Use of computer-aided drug design (CADD), computer-aided molecular modelling, pharmacophore modelling, molecular docking, quantitative structure–activity/property relationships and other calculation methods in discovery and development is continuously increasing (28). Therefore, the compilation and accessibility of the public contribution of this indigenous phytochemical and its derivatives can be used as a gem to decorate the crown of rational drug design.

## Construction and content

### Literature mining and data assembly

In the initial stage of the database construction, phytochemical data were manually compiled from numerous published documents. This process was carried out in google scholar (https://scholar.google.com/) and PubMed (https://pubmed.ncbi.nlm.nih.gov/) using specific keywords phytochemicals’, ‘compounds’, ‘plant-derived molecules’ with scientific names and local names of shortlisted plants. This search guides a list of scientific literature related to plant-derived phytochemicals.

A comprehensive review of 935 scientific papers from well-known journals was carried out to identify unique phytochemicals to build a powerful database based on phytochemicals. Journals such as ‘*Phytochemistry*’, ‘*Plant Medica*’, ‘*Journal of Medicinal Plant Research*, ‘*Journal of Natural Products*’ and ‘*Journal of Pharmaceutical Sciences*’ are the few sources of information reported in this database. Eight thousand and ninety-three unique phytochemicals from 525 plants were assigned to an extensive list of phytochemicals to construct the database. This library was developed using Microsoft Office and Excel software. Each phytochemical obtained from these documents has been passed through PubChem (https://pubchem.ncbi.nlm.nih.gov/), ChemSpider (http://www.chemspider.com/), ChEMBL (https://www.ebi.ac.uk/chembl/) and DNP (http://dnp.chemnetbase.com/) databases.

A separate phytochem ID was assigned to each phytochemical, creating a total of 8093 search entries for the website. In addition, 3D and 2D chemical structures were retrieved from the PubChem server to facilitate *in silico* drug discovery to find new phytochemical clues. The physicochemical properties of these phytochemicals such as molecular formula, canonical SMILES, molecular weight, no. of heavy atoms, no. of aromatic heavy atoms, fraction Csp3, no. of rotatable bonds, no. of H-bond acceptors, no. of H-bond donors, molar refractivity, TPSA, GI absorption, BBB permeant, P-gp substrate, CYP1A2 inhibitor, CYP2C19 inhibitor, CYP2C9 inhibitor, CYP2D6 inhibitor, CYP3A4 inhibitor, Log Kp, Ghose, Veber, Egan, Muegge, bioavailability scores, PAINS, Brenk, Leadlikeness, synthetic accessibility, iLOGP and Lipinski rule of five were retrieved from the SwissADME web tool (http://www.swissadme.ch/).

### Database web interface development

The web interface was developed using Django and PostgreSQL databases. Django is a free and open-source web framework based on Python. PostgreSQL is a free and open-source relational database management system. The web pages were rendered using HTML, CSS and Bootstrap from the Django server.

### Phytochemdb data access

Phytochemdb can be studied to capture phytochemical information in a variety of ways; it provides an efficient and simple text search tool with multiple search options. Users can query by using four simple information as keywords: (i) plant name, (ii) unique accession number (Phytochem ID), (iii) canonical SMILES and (iv) molecular formula.

## Utility and discussion

Natural products from various medicinal plants are the main source of novel chemical entities (NCEs) with the potential to lead to the budding new drug (29). The process of bringing a new drug to market is slow, time-consuming and costly (4). At the same time, the failure rate of drug development in high-throughput screening and clinical trials is heaping sorrow upon sorrow (30). In these circumstances, phytochemicals are gradually becoming the central choice as drug leads due to having a record of being safer than other chemical entities (29). So, it needs to provide a comprehensive repertoire for these plant-based NCEs. For this motivation, we tried this task, and we hope it will help to promote drug development.

### Database description

‘Phytochemdb’ has been developed as a free, open-access resource that uses computational methods to provide comprehensive information about the phytochemicals of medicinal plants. These phytochemicals were obtained through a large number of literature excavations and follow-up confirmation of various well-known chemical databases. The 3D structures combined with canonical SMILES were incorporated in the database for easy retrieval of phytochemistry-related information. This rich database collects physicochemical properties including molecular formula, molecular weight, no. of heavy atoms, no. of aromatic heavy atoms, fraction Csp3, no. of rotatable bonds, no. of H-bond acceptors, no. of H-bond donors, molar refractivity and TPSA. In addition, the evaluation of pharmacokinetics properties, including GI absorption, BBB permeant, P-gp substrate, CYP1A2 inhibitor, CYP2C19 inhibitor, CYP2C9 inhibitor, CYP2D6 inhibitor, CYP3A4 inhibitor and Log Kp (skin permeation), is also available in this database. Additionally, evaluations oriented towards the prominence of drug similarities, such as Lipinski rule of five, Ghose, Veber, Egan, Muegge and bioavailability score, are assembled in the database. In addition, a huge database features based on medicinal chemistry including PAINS, Brenk, Leadlikeness and synthetic accessibility and lipophilic properties like iLOGP. By searching a unique accession number (Phytochem ID), canonical SMILES, molecular formula and plant names, anyone can retrieve information about these compounds. One can download a composite set of specific plants and an entire data set of ‘phytochemdb’ through institutional e-mail. In addition to queries, the database also relates to the availability of each phytochemical in other plants. Those who are curious about CADD, lead optimization, virtual screening and molecular docking can benefit greatly from this assortment. The database is also open for contribution from some application conditions.

### Database web interface

**Figure 1.**
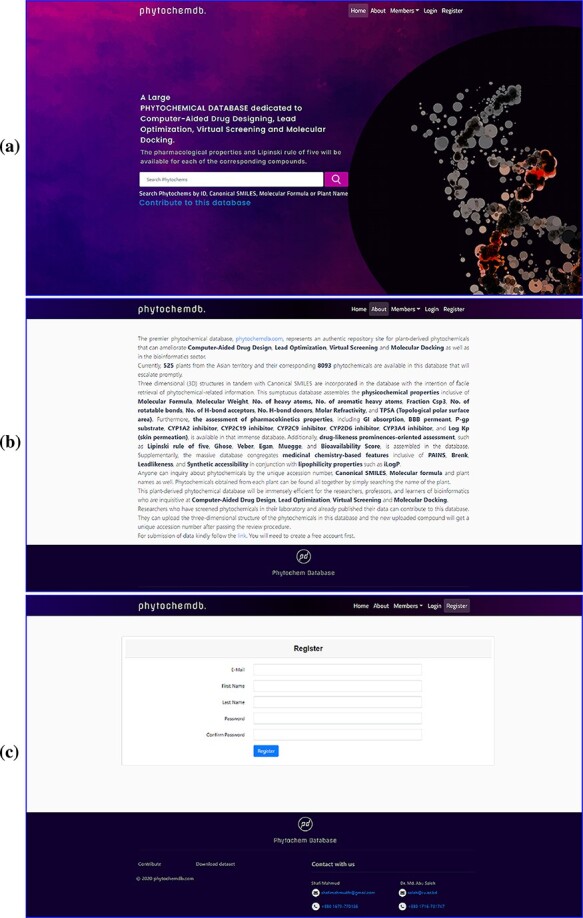
Web interface of the ‘phytochemdb’ database. (a) Demonstration of homepage containing the accessible search option. (b) ‘About’ web page encompasses a brief description about the database. (c) ‘Register’ web page comprehends the options for creating a free account in the database.

To build a well-defined database layout for quick and easy navigation, a simple navigation interface was developed. The ‘phytochemdb’ homepage interface contains ‘Home’, ‘About’, ‘Members’, ‘Login’ and ‘Registration’ options (Figure 1a). Here, the ‘About’ option provides some necessary information about the database (Figure 1b). Clicking on the ‘Member’ keyword will show the details of the contribution of researchers, developers and supervisors. The keyword ‘Register’ was catered for users to register for ‘phytochemdb’ account for free; it only requires a user name, e-mail ID, password and a subsequent email activation (Figure 1c).

**Figure 2.**
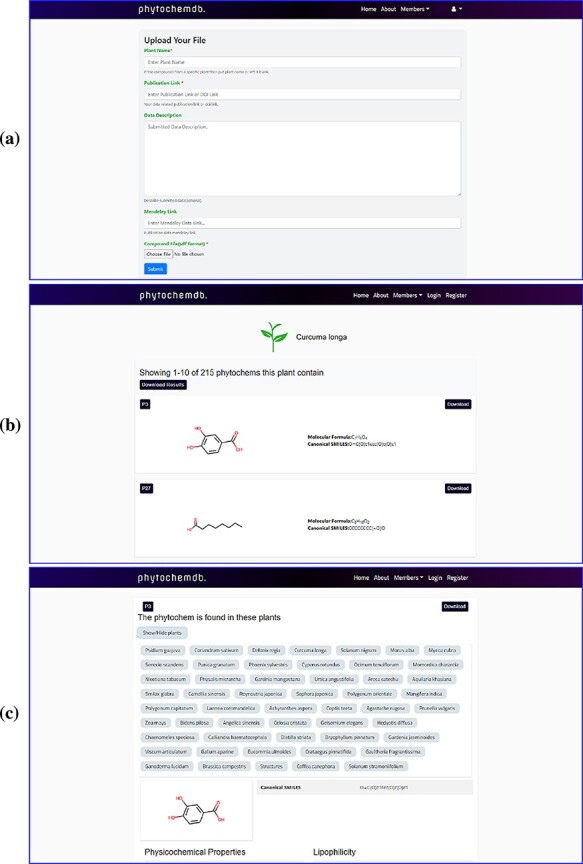
Web-interface of the ‘phytochemdb’ database. (a) Webpage for contribution to the database. (b) Webpage with all available compounds for a specific plant. (c) Webpage with relative availability of a compound in distinct plants.

After completing the registration process, members can log in with their e-mail ID and password. On the downhill slope of the homepage, members are provided with an open ‘Contribution’ option. Researchers who have screened phytochemicals in the laboratory and have reported their findings are welcome to contributions to the database. The submitter must present the plant name, publication link, compound structure in SDF as well as other information (Figure 2a). After approval, the newly uploaded compound will be assigned a unique accession number (Phytochem ID).

Users can only download the entire data set of this database in SDF format by clicking the ‘Download dataset’ option after registering through the organization’s email. There are many ways to explore ‘phytochemdb’ to retrieve phytochemical data; a simple text search tool is provided on the homepage, which provides multiple search options. Users can query through four simple information, such as (i) plant name, (ii) unique accession number (Phytochem ID), (iii) canonical SMILES (iv) and molecular formula. If the user browses for compounds through the plant name, the result page will display the list of all available compounds for that specific plant (Figure 2b).

In addition to querying plants, the availability of these compounds in other plants will also be shown. Users will be able to download the complete phytochemical data set of the query plants or download them one by one according to their preferences. Or, if the user uses other search options such as phytochem ID, canonical SMILES and molecular formula, information of that specific compound will be displayed on the results page. By clicking on the result phytochem ID on the results page, a new page will be displayed describing information about the search compound. This page shows the relative availability of the compound in various plants (Figure 2c).

**Figure 3.**
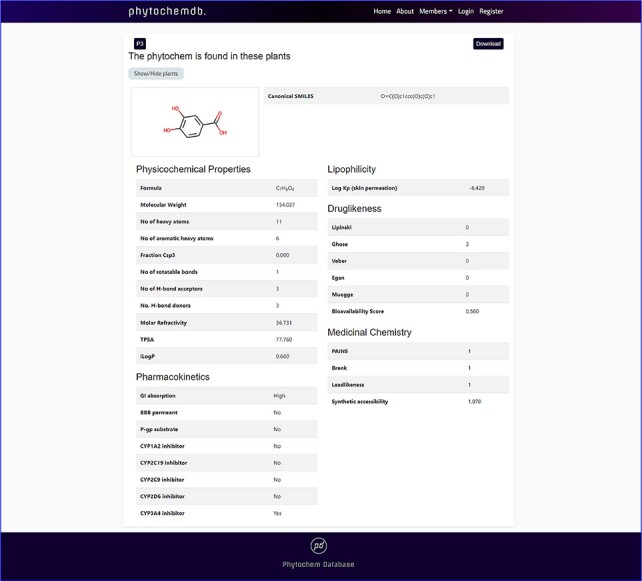
Features of ‘phytochemdb’ database encompassing the physicochemical properties of phytochemicals.

By clicking the ‘Download’ keyword, one will be able to download the 3D structure of the compound. Detailed information including physicochemical properties like molecular formula, canonical SMILES, molecular weight, no. of heavy atoms, no. of aromatic heavy atoms, fraction Csp3, no. of rotatable bonds, no. of H-bond acceptors, no. of H-bond donors, molar refractivity, TPSA, GI absorption, BBB permeant, P-gp substrate, CYP1A2 inhibitor, CYP2C19 inhibitor, CYP2C9 inhibitor, CYP2D6 inhibitor, CYP3A4 inhibitor, Log Kp, Ghose, Veber, Egan, Muegge, bioavailability scores, PAINS, Brenk, Leadlikeness, synthetic accessibility, iLOGP and Lipinski rule of five with the number of violations will be displayed down to the page (Figure 3).

### Database statistics

This database currently contains 525 plants and their corresponding 8093 phytochemicals, and it is expected to increase rapidly. The physicochemical properties of the compounds can be obtained from the ‘phytochemdb’ database, which has important value in determining the similarity of drugs.

**Figure 4.**
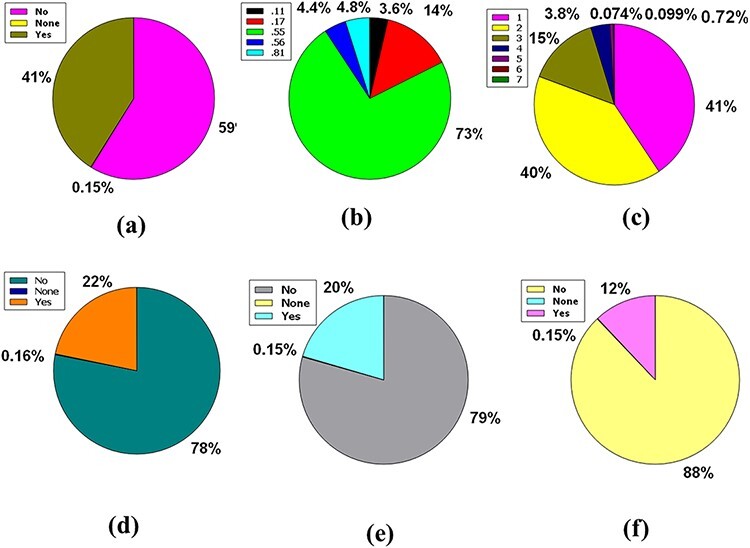
Venn diagram plot illustration of the physicochemical properties of phytochemicals in ‘phytochemdb’ database: (a) BBB permeable and impermeable proportions of phytochemicals; (b) percentages of phytochemicals that satisfy diverse bioavailability scores; (c) the Brenk structural alert of compounds with different percentages; proportions of (d) CYP1A2, (e) CYP2C9, (f) CYP2C19 inhibitors and non-inhibitors of phytochemicals in the database.

The central nervous system (CNS) is vascularized through some blood veins. These blood vessels are wrapped in an unparalleled feature called the blood–brain barrier (BBB). It conforms to these veins to hermetically control the movement of molecules, ions and cells within the brain and blood (31, 32). The BBB prevents most drugs from getting into the brain from the blood. Due to the existence of BBB (33), various radiopharmaceuticals used for brain neuroimaging or modern brain disease therapy have become tenacious. Fifty-nine percent of the compounds included in the full database are impermeable to BBB, but 41% of the compounds are permeable, along with the remaining 0.15%, which caused neither condition (Figure 4a).

The bioavailability score or ABS must be 0.55 to perform as a viable oral drug (34, 35). Notably, 73% of the compounds in the database had a bioavailability score of 0.55, while 14%, 4.8%, 4.4% and 3.6% of the compound accounted for 0.17, 0.81, 0.56 and 0.11 bioavailability scores indicate that >73% compound of the database provides a drug probability that matches bioavailability (Figure 4b).

Brenk understands a deep structural warning regarding the collection of chemical moieties including dye, unstable, toxic and more (36–38). Forty-one percent and 40% of contemporary compounds in the database displayed consecutive warnings of 0 and 1, while compounds of 15% and 3.8% of compounds displayed consecutive warnings of 2 and 3. In addition, skimpy percentages such as 0.72%, 0.074% and 0.0999% compound bids in the database consistently reported 4, 5 and 6 Brenk warnings (Figure 4c).

Cytochromes P450 (CYPs) are a super family of five major basal isoenzymes (CYP1A2, CYP2C19, CYP2C9, CYP2D6 and CYP3A4), which are involved in the elimination of the drug by metabolic biotransformation. The limited property of CYP is regulated by specific pharmacokinetics drug (39, 40). Seventy-eight percent of the purchasable compounds in the database appeared to be non-inhibitors of CYP1A2, while 22% of the compounds were concomitant inhibitors of CYP1A2 with 0.16% of a complex in the non-partition (Figure 4d).

According to the premise of CYP2C9 inhibition, 79% of compounds of the database are CYP2C9 non-inhibitor, whereas 20% are CYP2C9 inhibitors with 0.15% consolidated in none (Figure 4e). Furthermore, the vast database unifies 88% of CYP2C19 non-inhibitors, as well as 12% and 0.15% of compounds classed as CYP2C19 inhibitors or non-categorized, respectively (Figure 4f).

**Figure 5.**
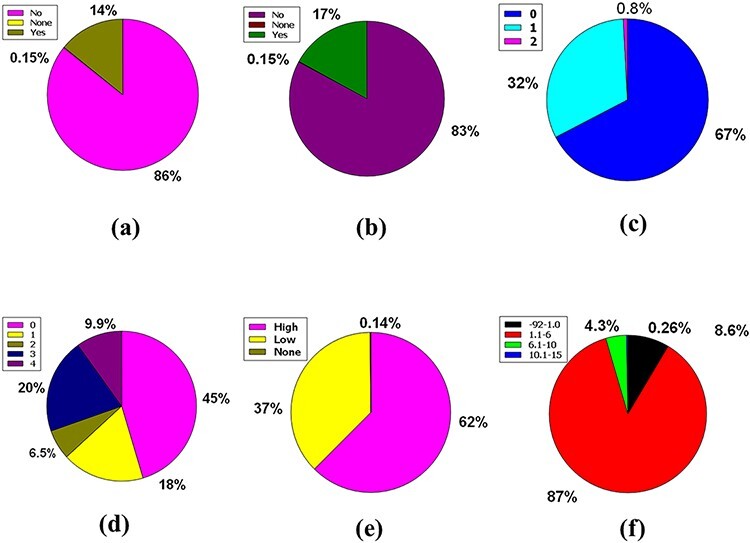
Venn diagram plot demonstration of the physicochemical properties of phytochemicals in ‘phytochemdb’ database: percentages of (a) CYP2D6, (b) CYP3A4 inhibitors and non-inhibitors of phytochemicals; (c) proportions of compounds that fulfil diverse criteria of Egan rule; (d) percentages of phytochemicals according to the Ghose filter; (e) gastrointestinal (GI) absorption rate of compounds; (f) percentages of phytochemicals that satisfy diverse iLOGP (lipophilicity) score.

Likewise, 86% of compounds in the database were not inhibitors of CYP2D6, while 14% were inhibitors of CYP2D6, plus 0.15% were classed as none (Figure 5a). So, the huge database added 83% of compounds of the database are CYP3A4 non-inhibitor, 17% compounds are CYP3A4 inhibitors, and 0.15% compounds are non-categorized (Figure 5b).

Egan’s rule contains two criteria: TPSA allowing TPSA ≤130 Å^2^ appraisal and logP requiring −1.0 ≤ logP ≤ 5.8 purviews (41, 42). Sixty-seven percent of compounds of the database appease the TPSA and logP inference pair. Furthermore, 32% of the compounds violate a single requirement, either TPSA or logP. Nonetheless, a compound of 0.8% perpetuates two violations of the Egan rule, implying an inability to carry out the two TPSA and logP dicta (Figure 5c).

Benempt Ghose filter is a new additional drug-likeness criterion that convenes four aphorisms including 160 ≤ molecular weight (MW) ≤ 480, −0.4 ≤ WLOGP (lipophilicity) ≤ 5.6, 40 ≤ molar refractivity (MR) ≤ 130 and 20 ≤ atoms ≤ 70 (43, 44). Notably, 45% compounds of the database exhibit zero violations when considering Ghose filter. One, two, and three criteria concerning the Ghose filter are violated by 18%, 6.5%, and 20% compounds of the database, respectively. However, 9.9% of compounds prevailing in the database displayed four violations because of skipping the specifications of the Ghose filter (Figure 5d).

Additional pharmacokinetic behavior confirmation for oral ingestion necessitates an appropriate GI absorption rate measurement (45,46). A high GI absorption rate accounts for 62% of the compounds, while a low GI absorption rate accounts for 37%, with 0.14% of the compounds falling into neither group. As a result, according to the GI absorption rate, % of the material is drug-like (Figure 5e).

The n-octanol/water apportionment coefficient, also known as log Po/w or partition coefficient (iLOGP), is a well-known physicochemical schema with an optimal range of −3.93 to 6.46, alluding to the extent of estimated log Po/w standard according to a drug-likeness criterion (47, 48). The database contains 1.1–6.00 iLOGP compass, which accounts for 87% of compound tenanting. In the database, 8.6%, 4.3% and 0.26% of the compounds are grouped into −92.0 to 1.00, 6.1–10.0 and 10.1–15.0 iLOGP expanses, successively (Figure 5f).

**Figure 6.**
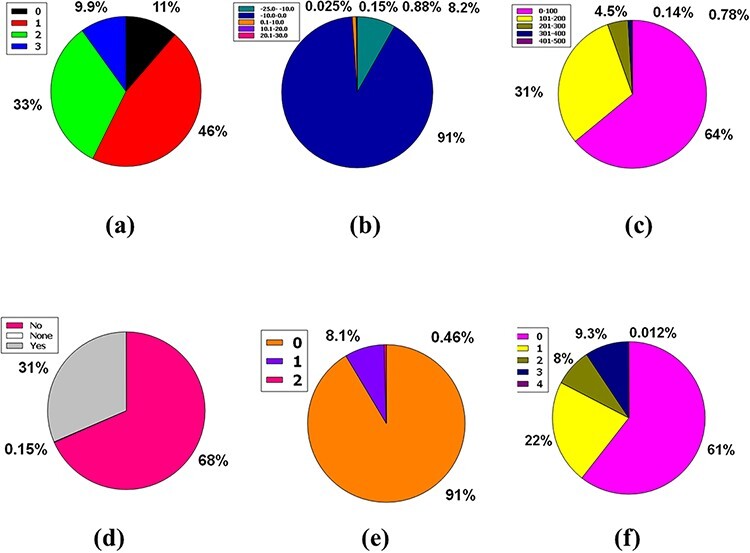
Venn diagram plot exhibition of the physicochemical properties of phytochemicals in ‘phytochemdb’ database: (a) proportions of compounds Leadlikeness; (b) LogKp percentages of compounds; (c) percentages of phytochemicals that satisfy diverse molar refractivity score; (d) P-gp substrate and P-gp non-substrate proportions of phytochemicals; (e) proportions of compounds that accomplish diverse standards of PAINS alert; (f) percentages of phytochemicals that satisfy diverse criteria of Lipinski rule of five.

The three premises of Leadlikeness, a well-known medicinal chemistry phrase, are as follows: 250 ≤ Molecular Weight ≤ 350, XLOGP ≤ 3.5 in conjunction with the number of rotatable bonds ≤ 7 (49). 11% of the database’s compounds have zero violations, while 9.9% have neither of the Leadlikeness requirements. Approximately 46% and 33% of the database’s compounds have one or two Leadlikeness violations, respectively (Figure 6a).

The skin permeability coefficient or Kp in cm/s unit is prognosticated by a pharmacokinetics prominence known as log Kp, which asserts that the more negative the log Kp, the lower skin permeant is the molecule and the standard compass for the skin permeability is −8.0 to −1.0 (39, 50). Notably, 91% of compounds found in the database are incorporated into the LogKp range of −10.0 to 0. Meanwhile, 8.2%, 0.88%, 0.15%, and 0.025% of the database’s compounds fall into the 25.0 to 10.0, 0.1 to 10.0, 10.1 to 20.0, and 20.1 to 30.0 LogKp ranges (Figure 6b).

It is understood that the acceptable range of molar refractivity (which is a measurement of the steric factor) is between 40 and 130, with a mean value of 97 (38, 43). In a database, 64% of compound tenanting exists in the molar refractory range of 0–100. However, in the molar refractory range of 101–200, the 31% of compounds dominate. Despite these, 4.5%, 0.78% and 0.14% of the compound of the database squats in the molar refractivity compass of 201–300, 301–400 and 401–500, respectively (Figure 6c). It is evident that a qualifying spectrum of drug-likeness according to molar refractivity exists in ≥64% of the compounds in the database.

The CNS or central nervous system is protected from distinct xenobiotics by the clepen P-gp (permeability-glycoprotein) substrate, whose overexpression leads to multidrug-resistant malignancy (39, 51). Around % of the database’s compounds are P-gp non-substrate, 31% are P-gp substrate, and only 0.15% are neither P-gp substrate nor P-gp non-substrate (Figure 6d). As a result, ∼68% of the compounds in the database satisfy the drug-likeness proviso following the pharmacokinetics baptized P-gp substrate.

PAINS is a word that has grown in use to describe identifiable bioactive substances that are difficult to detect in readouts due to interconnection with uncovenanted biological objects and procedures. The rationale for the name PAINS is that it stands for sluggish drug improvement choices, and to eliminate incorrect results, PAINS must have kenned and staved off (52–54). In this massive database, 91% of compounds have no PAINS alert, while 8.1% and 0.46% of compounds had one and two PAINS alerts, respectively (Figure 6e).

The Lipinski rule of five consists of four conditions that must be met for a drug-alike compound to satisfy the Lipinski rule. According to the Lipinski rule, drug-like compounds must have a logP standard of <4.15 (MLOGP ≤ 4.15), which represent their hydrophobicity, a hydrogen bond donor of <5 (NH or OH ≤ 5), a molecular weight of <500 g/mol (MW ≤ 500) and a hydrogen bond acceptor of <10 (N or O ≤ 10) (55). There are 61% of compounds that do not break any Lipinski criteria. Furthermore, 22% of the database compounds violate at least one of these requirements, while 8% of compounds violate two rules. However, 9.3% and 0.012% of the compounds in this plenary database violate 3 and 4 rules of the Lipinski rule of five, respectively (Figure 6f).

**Figure 7.**
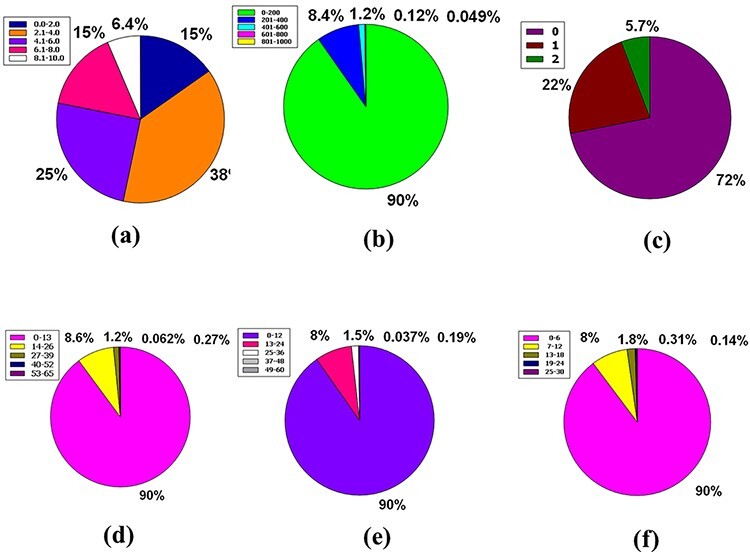
Venn diagram plot display of the physicochemical properties of phytochemicals in ‘phytochemdb’ database: (a) proportions of compounds’ synthetic accessibility; (b) proportions of phytochemicals that show diverse standards of TPSA; (c) percentages of phytochemicals according to the Veber filter; (d) proportions of compounds that fulfil different standards of rotatable bonds; (e) and (f) percentages of phytochemicals that satisfy various criteria of H bond acceptors and H bond donors, respectively.

Accessibility in a synthetic form calculating the ease of synthesis is planned, with a score ranging from 1 to 10, where 1 denotes easy to make and 10 denotes difficult to make (56, 57). Fifteen percent of compounds are included in the 0.00–2.00 ranges, while 38% are included in 2.1–4.00 ranges. Furthermore, 25%, 15% and 6.4% of compounds in the database have coalesced in the 4.1–6.00, 6.1–8.00 and 8.1–10.00 compass in that order (Figure 7a).

For drug-likeness, the usual level of TPSA explains a range of 0–140 (58, 59). This plenary database is stocked with similar compounds that account for 90% of the compound with a favourable TPSA range such as 0–200. The remaining 10% of compounds have a wide range of videlicet, including 201–400, 401–600, 601–800 and 801–1000, which envelop 8.4%, 1.2%, 0.12% and 0.049%, respectively (Figure 7b).

In terms of the Veber rule, compounds with a polar surface area ≤140 Å^2^ (TPSA ≤ 140) and 10 or more rotatable bonds (rotatable bonds ≤10) have a high probability of positive oral bioavailability for drug-like candidates (60, 61). Both of these requirements are met by a large number of chemicals (72%) in the database.

Furthermore, 22% of compounds could only match one of these two criteria, although just 5.7% of the total number of compounds in our database could not meet either of these criteria (Figure 7c).

The number of rotatable bonds (RB) in a molecule that is less than 10 (RB10) (58) is used to determine its superior drug candidacy. 90% of the compounds in the database have rotatable bonds in the range of 0–13. However, the entire database includes a variety of rotatable bond ranges such as 14–26, 27–39, 40–52 and 53–65, which correspond to 8.6%, 1.2%, 0.27% and 0.062% of compounds, sequentially (Figure 7d).

Hydrogen bond acceptor is a critical criterion when it comes to a drug-alikeness molecule, indicating that no ≥10 hydrogen bond acceptors are preferred for the drug-likeness feature (62, 63). Although the range interim (13–24) has a humilis assessment of compounds of 8%, this database has a significant share of 90% of compounds with hydrogen bond acceptors inside 0–12. Furthermore, the database has a specific range of hydrogen bond acceptors with a significantly lower proportion, such as 25–36 is 1.5%, 37–48 is 0.19% and 49–60 is 0.037% (Figure 7e).

For a favourable drug candidate, hydrogen bond donors should be <5 (62, 63). Ninety percent of compounds of the entire database had a hydrogen bond donor ranging from 0 to 6. But compounds that include the range of 7–12 represent 8% of the entire database. In addition, the thin segment of this database includes 1.8%, 0.31% and 0.14%, containing many clear ranges of hydrogen bond donors, namely 13–18, 19–24 and 25–30, sequentially (Figure 7f).

**Figure 8.**
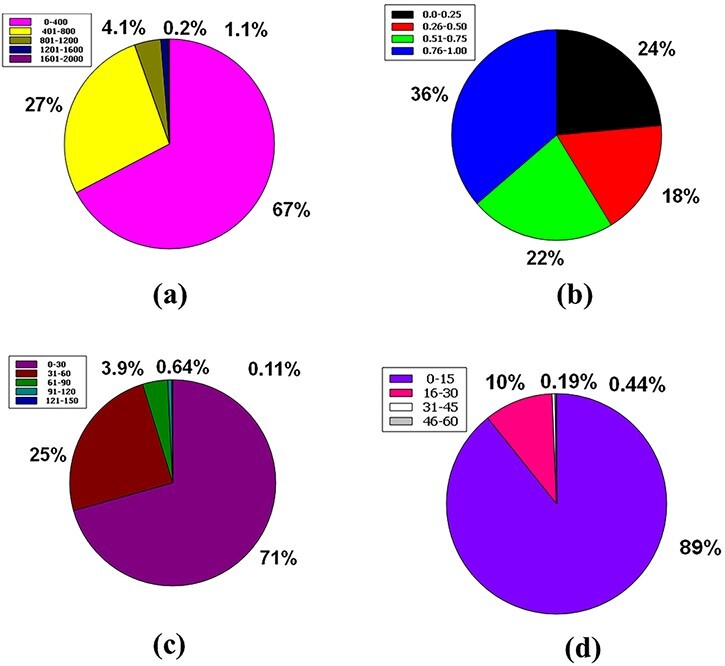
Venn diagram plot illustration of the physicochemical properties of phytochemicals in ‘phytochemdb’ database: (a) proportions of compounds molecular weight; (b) percentages of phytochemicals that satisfy diverse fraction Csp3 score; (c) and (d) percentages of phytochemicals that fulfil various criteria of heavy atoms and aromatic heavy atoms, respectively.

To be a drug, the molecular weight of a drug compound should be < 500 g/mol (MW ≤ 500 g/mol) (55, 64). In the database, 67% of the molecular weight of the compound’s molecular weight is in a compass of 0–400 g/mol. Then, 27% of the compounds are in the range 401–800 g/mol. 4.1% of compounds had a molecular weight ranging from 801 to 1200 g/mol. Ultimately, 1.1% and 0.2% of compounds were between the molecular weight ranges from 1201 to 1600 g/mol and from 1601 to 2000 g/mol, respectively (Figure 8a).

The ratio of sp3 hybrid carbons to the total carbons of the molecules (Fraction Csp3) should be 0.25 to <1 (39, 65). Here, 36% of the raw database is covered with a Csp3 fractional interval of 0.76–1.00, while 24% is in the range of 0.0–0.25. The next 22% phytochemicals of this database have another range of 0.51–0.75, and finally, 18% phytochemicals have a range of 0.26–0.50 (Figure 8b).

There have been no experiments carried out to investigate the simultaneous effect of the mentioned portion of heavy atoms on the drug-like feature of any compound (66). Nonetheless, the database has information regarding heavy atoms in specific molecules. Around 71% phytochemicals of the database are made up of heavy atoms ranges between (0–30), whereas 25%, 3.9%, 0.64% and 0.11% phytochemicals contain heavy atoms ranges from (31–60), (61–90), (91–120) and (121–150), respectively (Figure 8c).

No study continuously studies the simultaneous oscillation of aromatic ring numbers to include the drug similarity characteristics of the compound (66). Eighty-nine percent (89%) of the entire database contains compounds with aromatic heavy atoms between 0 and 15. Approximately 10% compounds of the database have aromatic heavy atoms ranging from 16 to 30. Furthermore, 0.44% and 0.19% compounds of this database contain aromatic heavy atoms with a range of 31–45 and 46–60, respectively (Figure 8d).

### Comparison between ‘phytochemdb’ and other databases

Since natural products have become the centre of attention of the scientific community in the last few decades, various well-known databases such as PubChem, Zinc, ChEBI, ChEMBL, ChemBridge, ChemSpider and DrugBank have been constructed (4, 19). These databases contain millions of compounds, but none of them are particularly relevant to medicinal plants. In the past few decades, several medicinal plants databases have been developed, such as MAPS, HerbMed, MPDB 1.0, GLOBinMED, MEDDB, phytochemia and IMPPAT. However, these databases have some key limitations (19). Some of these databases are only useful to certain areas, while others just offer the ability to download compounds, and still others lack critical physicochemical data. Furthermore, the quality of molecular structure of compounds in some of these databases is not fully understood. Furthermore, some of these databases aren’t updated or maintained on a regular basis (Table 1). Chem-TCM is a digital database that contains 12070 chemical compounds from 350 plants that are commonly utilized as traditional Chinese herbal medicine. This database includes chemical compounds with a variety of chemical–physical properties, such as molecular formula, molecular weight, canonical SMILES, natural product class, scaffold, logP, number of rotatable bonds, number of rings, number of aromatic rings, number of hydrogen bond acceptors, number of hydrogen bond donors, polar surface areas, and chirality (http://chemtcm.com/). Besides, TIPdb is a constructed and explorative database of anti-cancer, anti-tuberculosis and anti-platelet phytochemicals from homespun plants in Taiwan. Physiochemical properties of this database subsume hydrogen bond acceptors, hydrogen bond donors, rotatable bonds, TPSA, molecular weight and XLOGP (https://cwtung.kmu.edu.tw/tipdb/index.php). Compared with other phytochemical databases, ‘phytochemdb’ covers most of the functions of the phytochemical database on a single platform. It gathers almost all necessary information such as molecular structure, molecular weight and physiochemical properties and provides a simple phytochemical navigation platform that can improve CADD, lead optimization, virtual screening, molecular docking and bioinformatics.

**Table 1.** Comparison between ‘phytochemdb’ and other databases

Database name	Natural product type	Number of phytochemicals with correct structure	Freely accessible	Requires a registration	Is maintained (2019)	Is updated	Most recent publication (DOI)
Phytochemdb	Traditional medicine, plants	8093	Yes	Not compulsory	Yes	Yes	x
AfroDB	Traditional medicine, plants, Africa	954	Yes	No	No	No	https://doi.org/10.1021/ci300 309k
Ayurveda	Traditional medicine, plants, Asia	950	No	Yes	Yes	Unknown	https://doi.org/10.18097/PBMC20156102286
Chem-TCM	Plants, Traditional medicine, Asia	12 070	No	Yes	Yes	No	https://doi.org/10.1016/j.bmc.2010.01.070
ChemDB	Plants, Asia	> 1000	Unknown	Unknown	No	No	https://doi.org/10.2174/157340991102150904101740
HIM	Drug-like, traditional medicine, plants	1261	Yes	No	No	No	https://doi.org/10.1186/1758-2946-5-28
NADI	Traditional medicine, plants	3000	No	Yes	Yes	Unknown	https://doi.org/10.1021/ci500405g
Phytochemica	Plants, traditional medicine, Asia	571	Yes	No	No	No	https://doi.org/10.1093/database/bav07 5
TCMSP	Plants, Traditional medicine, Asia	29 384	Yes	No	No	No	https://doi.org/10.1186/1758-2946-6-13
TIPdb	Asia, plants, drug-like	13 177	Yes	No	Yes	No	https://doi.org/10.1093/database/bau05 5
UEFS	Plants, America	503	Yes	No	No	No	x

### Future prospective

We look forward to expanding the database by including more phytochemical data from a large number of medicinal plants. We also plan to use some reliable calculation tools to provide predicted interactions between phytochemicals and human target proteins in our database. For each phytochemical molecule, we are attempting to offer predicted ADMET properties. As a result, the database will be updated to include new chemical and pharmacological properties that can be used to predict more drug similarity properties. Bioinformaticians can use these techniques to create useful tools for drug design and virtual screening workflow. Other scientific fields, such as pharmaceutical chemistry and molecular biology, can benefit from the species and phytochemical contents. The availability of phytochemical data on a single platform will allow fellow researchers to access information in real time. There are also options for importing data from other research groups, which will help to broaden the data set. Phytochemical filtering options will be provided in near future based on their physicochemical and druggable properties.

## Conclusion

Medicinal plants are considered an important natural source for discovering novel drugs and therapeutics. Appropriate resources on these plants integrate important information such as chemical structure, pharmacology and physiochemical properties. They are reasonably laid out and easy to navigate. They will become assets of computational pharmacology and drug discovery. ‘phytochemdb’ is built on this motto and hopes it will enable people to effortlessly pursue knowledge of medicinal plants and active ingredients. Its rich collection and convenient accessibility will help researchers and the pharmaceutical industries that are curious about medicinal plants. Since the path of drug discovery and new drug launches is built on failed cobblestones, ‘phytochemdb’ can help to speed up the drug development process by saving time and money and follow the pharmaceutical company’s mantra ‘fail fast, fail early’.

## Key points

- Presents ‘phytochemdb’ a structured compilation of 525 plants and their corresponding 8093 phytochemicals.
- Details the FDA drug candidacy acceptance criteria.
- ‘Phytochemdb’ covers the limitations of previously developed databases.
- Focuses on rich collection and convenient accessibility for searching potential druggable targets for the treatment of chronic diseases.
